# Characteristics of symptomatic men with testicular microlithiasis – A Danish cross‐sectional questionnaire study

**DOI:** 10.1111/andr.12326

**Published:** 2017-03-07

**Authors:** M. R. Pedersen, H. Møller, S. R. Rafaelsen, M. M. B. Jørgensen, P. J. Osther, P. Vedsted

**Affiliations:** ^1^ Department of Radiology Vejle Hospital Part of Lillebaelt Hospital Vejle Denmark; ^2^ Urological Research Centre Vejle Hospital Part of Lillebaelt Hospital Vejle Denmark; ^3^ Institute of Regional Health Research University of Southern Denmark Odense Denmark; ^4^ Cancer Epidemiology and Population Health King's College London London UK; ^5^ Research Unit for General Practice Department of Public Health Aarhus University Aarhus Denmark; ^6^ Department of Clinical Medicine Aarhus University Aarhus Denmark

**Keywords:** Denmark, questionnaire, testicular microlithiasis, testis cancer, ultrasound examination

## Abstract

Testicular microlithiasis (TML) is an incidental finding at ultrasonography of the scrotum. A link between testicular microlithiasis and testicular cancer has been suggested. However, the majority of studies are retrospective using ultrasonography with minor data on health status and life style characteristics. Our objective was to investigate if lifestyle and health are associated with TML. In 2014, we conducted a self‐administered questionnaire survey including 1538 men, who all due to testicular/scrotal symptoms had an ultrasound investigation of the scrotum during 2004–2013. The men were divided into men with TML and men without. The 23‐items questionnaire included items on age, height, weight, lifestyle (alcohol consumptions, smoking habits, workload, exercise and food), previous diseases in the testicles, pain and consumption of analgesics. The prevalence of TML was 12.8%. Overall, lifestyle factors did not vary between men with or without TML. However, men with TML did consume more crisp than men without. Development of TML was not associated to classic life style factors such as alcohol consumption, smoking habits, or mothers smoking during pregnancy. Also, age and height could not be linked to presence of TML. We did find, however, that men with TML experienced less physical activity and consumed more crisp than men without TML. Since ingestion of crisps has potential carcinogenic effect (acrylamide), this finding needs confirmation in a separate study.

## Introduction

Testicular microlithiasis (TML) is as an incidental finding by ultrasonography (US) of the scrotum. TML was described by Doherty *et al*. (1987) as innumerable tiny bright echoes diffusely and uniformly scattered throughout their substance. The incidence of TML has increased with the development of more high‐end ultrasound machines, and perhaps also increased awareness of diagnosis of testicular cancer. TML prevalence varies considerably in studies of asymptomatic men (0.6–9.0%) (Derogee *et al*., 2001; Miller & Sidhu, 2002; Kim *et al*., 2003; Goede *et al*., 2009) compared to symptomatic (e.g. pain, lump or swelling) men (8.7–18.1%) (Bach *et al*., 2001; Middleton *et al*., 2002; Deganello *et al*., 2012). In populations with genetic disorders, the prevalence of TML has been reported as high as 17.5% in men with Klinefelter syndrome (Accardo *et al*., 2015) and 36.0% in men with Down's syndrome (Cebeci *et al*., 2015).

TML has been associated with carcinoma in situ and testicular cancer (Derogee *et al*., 2001; Sakamoto *et al*., 2006); however, not all studies have found this association (Peterson *et al*., 2001; Costabile, 2007; DeCastro *et al*., 2008; Goede *et al*., 2009; Accardo *et al*., 2015). A recent meta‐analysis including more than 35 000 men concluded that TML was significantly associated with risk of testicular cancer (Wang *et al*., 2015). Still, it is not evident whether there is a causal relationship or the association is a result of confounding by indication. In 2015, the first guideline was published by the European Society of Urogenital Radiology (ESUR) recommending yearly US follow‐up in men with TML and additional risk factors (personal/family history of testicular cancer, maldescent, orchidopexy, atrophy or genetic disease) (Richenberg *et al*., 2015). The risk factors for testicular cancer are not clear, but there are some acknowledged or suggested risk factors for example, cryptorchidism (Cook *et al*., 2010), height (Wiren *et al*., 2014), family history (Nordsborg *et al*., 2011) and maternal smoking (Pettersson *et al*., 2004). The majority of TML studies are retrospective with limited background information. Thus, there is a need for more knowledge about lifestyle and health in men with TML, in order to understand if lifestyle influences development of TML.

The aim of this study was to investigate if lifestyle and health were associated with TML.

## Materials and Methods

A 23‐item self‐administered questionnaire was in 2014 mailed to men who all had undergone an US investigation of the scrotum in a 10‐year period. After returning the questionnaires, the men were categorised into men with or without TML, based on the result of their previous US. The questionnaire was explicitly developed to gain more knowledge about TML.

### Study population

All men who had an US investigation of the scrotum in our radiology department between 01.01.2004 and 31.12.2013 were identified in the Roentgen Information System (RIS), which included patient's unique civil registration number (Lynge *et al*., 2011), referral, date of investigation and the medical US conclusion. The civil registration number was used to collect information in biopsies and cancer subtype when relevant.

**Figure 1:**
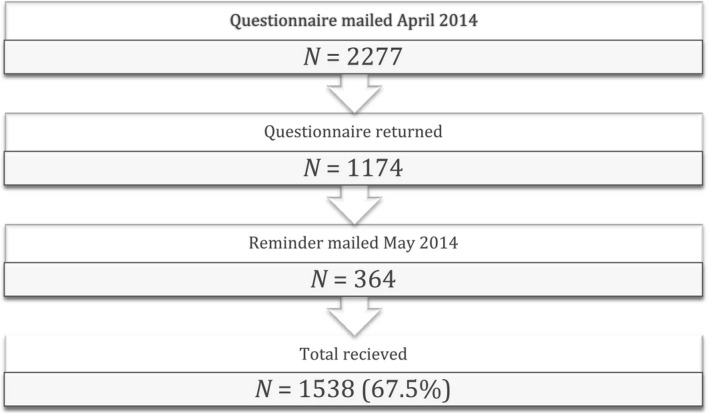
Flow chart of the questionnaires.

A total of 2610 men were identified. Because of death, unknown address, having left the country, under the age of 18 years at the date of investigation, a total of 2277 men were mailed a questionnaire. A total of 1214 (53.3%) questionnaires were returned, including 21 who did not wish to participate, 11 with unknown address and eight returned an unidentified questionnaire and were excluded, leaving 1174 questionnaires eligible (51.6%). A reminding letter was mailed after four weeks and further 369 questionnaires were returned, of which five individuals did not want to participate. This left us with a total of 1538 questionnaires (67.5%) (Figure 1).

All the questionnaires were entered into two databases separately by two of the authors (MRP and MMJ), and compared in order to identify any discrepancies, which were then revised.

All US examinations were performed by one of three scanners; either a 9L4 linear array transducer from Siemens (Siemens, Acuson, S2000, Mountain View, CA, USA/Siemens, Acuson, S3000) or with 14‐6 linear array transducer from Hitachi (Hitachi, EUB‐8500, Tokyo, Japan).

### Questionnaire

The 23 items included questions about age, height, weight and lifestyle items on food and alcohol consumptions, smoking habits, exercise, previous diseases in the testicles and consumption of analgesic. Multiple answers for a single item were not allowed.

The questionnaire was developed to describe the concepts of health and lifestyle among the men. It included a broad area of both self‐reported experience of testicular symptoms, a broad number of food and drink consumptions and personal characteristics. Items related to testicular cancer, frequently presented symptoms by patients referred to US investigation of the scrotum and ad hoc items were also included.

The patients were asked, how often they consumed a broad various of food and drink with response categories ‘more than once a day’, ‘5–7 times a week’, ‘3–4 times a week’, ‘1–2 times a week’, ‘1–3 times a month’ and ‘rarer’. In the analyses, these were dichotomised into ‘more than once a week’ and ‘less than once a week’ with the latter including all other response categories than ‘5–7 times a week’, ‘3–4 times a week’ and ‘1–2 times a week’.

To establish content validity in the questionnaire, four radiologists commented on the included aspects, words or formulations. The radiologists were all skilled in US, and all dealt on a daily basis with US investigation of the scrotum. In addition, we performed a pilot study among five patients, after they had completed an US investigation of the scrotum. Afterwards, one author (MRP) interviewed each patient concerning content and comprehension of the questionnaire, answer categories, length of questionnaire and time consumption.

After revision, a second pilot study was performed, among 26 randomly selected men after they had undergone an US of the scrotum. They filled out the 23‐item questionnaire and orally commented on it to one of the authors (MRP). Based on the pilot questionnaire, we adjusted one question (question 20) and added an extra disease category. In the pilot, we assessed the data for missing data and floor and ceiling effects, and adjusted the answer categories.

### Statistical analysis

The data were analysed using stata (version 14.1, STATA Corporation, College Station, TX, USA). The 95% confidence intervals (95%CI) for odds ratios (OR) were calculated by logistic regression and *p*‐values ≤5% were considered statistically significant.

### Ethics and approval

The study was approved by the National Data protection Agency, and the Regional Scientific Ethic Committee for Southern Denmark (ID: S‐20120144) and the national board of health.

## Results

In the identified study population of 2277 men, a total of 270 had TML (prevalence of 11.9%). Among the 1538 responding men included in the analyses, 197 (12.8%) men had TML. The mean age in men with TML was 51.9 years (18–89) compared to 54.3 years (18–93) for men without TML (*p* = 0.056).

Overall, there were limited differences in health and lifestyle between men with or without TML (Tables 1 and 2). Men with TML reported to consume crisp and popcorn more often (36.6% vs. 22.0%, *p* < 0.001), reported less physical exercise (38.6% vs. 48.2%, *p* = 0.011), and experienced more often testicular discomfort (34.5% vs. 27.7%, *p* = 0.058) than men without TML. Trauma to the testicles was reported more often in men with TML than in men without TML (8.6% vs. 5.3%, *p* = 0.065). A total of 27.9% of the TML men reported their mother smoked during pregnancy compared to 23.1% of men without TML (*p* = 0.135).

**Table 1:** Information of age distribution and other characteristics of men with and without testicular microlithiasis (TML)

Variables	Men with TML		Men without TML		Age adjusted		
	*N* = 197	%	*N* = 1341	%	OR	L	H
Age							
0–29	11	5.6	94	7.0	0.63	0.31	1.25
30–39	31	15.7	168	12.5	0.99	0.61	1.60
40–49	53	26.9	284	21.2	1.00		
50–59	42	21.3	270	20.1	0.83	0.54	1.29
60–69	33	16.8	265	19.8	0.67	0.42	1.06
70+	27	13.7	260	19.4	0.56	0.34	0.91
Height ≥180 cm vs. <180 cm	108	54.8	672	40.1	1.12	0.82	1.53
BMI of 25–30 vs. <25	84	42.6	576	43.0	0.99	0.71	1.37
Smoking ever vs. no smoking	107	54.3	714	53.2	0.91	0.82	1.00
≥15 vs. <15 cigarette pack year	63	32.0	422	31.5	0.89	0.53	1.47
Mother smoking during pregnancy vs. no smoking	55	27.9	310	23.1	1.27	0.89	1.80
No alcohol consumption vs. alcohol consumption during the last 12 months	10	5.1	75	5.6	0.92	0.46	1.80
≥6 cups of coffee vs. <5 cups per day	51	25.9	346	25.8	1.02	0.72	1.45
≥3 cups of Tea vs. <2 cups per day	13	6.6	90	6.7	0.97	0.50	1.91
Idle work vs. lifting	79	40.1	491	36.6	1.15	0.76	1.75
>6 h vs. never sitting down during work hours	33	16.7	245	18.3	0.90	0.52	1.57
>6 h vs. never walking and standing during work hours	30	15.2	259	19.3	0.82	0.45	1.49
Sitting down ≥5 h vs. <5 h	34	17.2	196	14.6	1.27	0.84	1.91
30 min or less vs. never biking hours per day	33	16.7	195	14.5	1.18	0.76	1.83
Yes vs. no regular exercise	76	38.6	646	48.2	0.71	0.52	0.98
Yes vs. no testicular pain during the last 6 months	54	27.4	306	22.8	1.24	0.88	1.74
Yes vs. no testicular discomfort	68	34.5	372	27.7	1.29	0.93	1.79
Inflammation vs. never inflammation	38	19.3	227	16.9	1.20	0.82	1.77
Trauma vs. never trauma	17	8.6	72	5.4	1.58	0.90	2.75
Torsion vs. never torsion	10	5.1	41	3.1	1.58	0.78	3.24
Monthly vs. yearly use of painkillers	78	39.6	452	33.7	1.33	0.95	1.86
Use vs. no use of paracetamol	116	58.9	874	65.2	0.79	0.58	1.08

**Table 2:** Patient's soft drink and food consumption

Variables	Men with TML		Men without TML		Age adjusted		
	*N* = 197	%	*N* = 1342	%	OR	L	H
Soft drink with sugar							
More than once a week vs. less than once a week	103	52.3	709	52.9	0.89	0.64	1.22
Soft drink without sugar							
More than once a week vs. less than once a week	84	42.6	506	37.7	1.16	0.85	1.58
Cake, candy, Ice cream							
More than once a week vs. less than once a week	156	79.2	1037	77.3	1.04	0.71	1.53
Crisps, popcorn etc							
More than once a week vs. less than once a week	72	36.6	295	22.0	1.95	1.39	2.73
Fast‐food							
More than once a week vs. less than once a week	39	19.8	215	16.0	1.19	0.80	1.76
Meat							
More than 3 times a week vs. <3 times a week	144	73.1	966	72	1.01	0.72	1.42
Poultry							
More than 3 times a week vs. <3 times a week	55	27.9	327	24.4	1.11	0.79	1.58
Fish							
More than once a week vs. less than once a week	136	69.0	853	63.6	1.33	0.96	1.85
Vegetable/vegan dishes							
More than once a week vs. less than once a week	122	62.0	797	59.4	1.15	0.84	1.57

Table 3 shows the most common causes of referral to US investigation. Pain was most common in both groups of men (32.2% in men with TML vs. 27.1% in men without, *p* = 0.151).

**Table 3:** The referral reasons to ultrasonic investigation of scrotum in the two groups

Reasons for referral	Men with TML		Men without TML		*p*‐value
	*N*	%	*N*	%	
Testicular pain	63	32.2	363	27.1	0.151
Nodule/lump	41	20.9	257	19.2	0.585
Soreness	43	21.9	311	23.2	0.671
Swelling	23	11.8	234	17.5	0.044
Others	26	13.2	175	13.0	0.954
Total	197	100.0	1341	100.0	

Table 4 shows the difference between men with and/or without TML and the number of received US scans. Men without TML had less US investigations performed than men with TML. A total of 167 (84.7%) of the 197 men with TML were diagnosed with TML at their first US investigation. Leaving 30 of the TML men with a later diagnosis of TML with a median time from first US until TML diagnosis of 56.2 months (min‐max = 2.3–92.1, interquartile interval = 14.2–77.9).

**Table 4:** The number of US investigations the patients were referred to in the period 2004–2013

Number of US investigations	Men with TML		Men without TML	
	*N*	%	*N*	%
1	105	53.3	1095	81.6
2	55	28.0	186	13.9
3	17	8.6	41	3.1
4	6	3.0	12	0.9
5+	14	7.1	7	0.5
Total	197	100.0	1341	100.0

Seventy‐two (4.7%) of the 1538 responding men had a total of 112 biopsies [17 (23.6%) with and 55 (76.4%) without TML], giving a relative risk of having a biopsy of 2.0 (1.1–3.4) among men with TML. A total of 36 (32%) of the biopsies were of the contralateral testicle.

A total of 46 cancers were diagnosed in 43 men (2.8%) [11 men with TML (5.6%) and 32 men without TML (2.4%), OR = 2.4 (1.1–5.0), *p* = 0.011] (Table 5). The mean age in men with testicular cancer was 42.5 (21–85) years. A total of 29 seminomas were diagnosed, seminoma being the most common cancer subtype in both groups. Four of the 43 men developed a testicular cancer between 1 and 3 years after US investigation and two of these men had TML.

**Table 5:** Overview of the 46 malignancy biopsies in 43 men (three of the men had malignancy in both testicles)

Cancer type	Men with TML		Men without TML		Total
	*N*	%	*N*	%	
Seminoma	6	20.7	23	79.3	29
Carcinoma in situ	1	100.0	0	0	1
Teratoma	1	33.3	2	66.7	3
Leydig cell tumour	0	0.0	1	100.0	1
Adenomatoid tumour	0	0.0	3	100.0	3
B‐cell lymphoma	1	100.0	0	0.0	1
Germinal cell tumour	2	28.6	5	71.4	7
Lipoid sarcoma	1	100.0	0	0.0	1
Total	12	26.1	34	73.9	46

## Discussion

### Main findings

In general, there were limited differences in health and lifestyle among men with or without TML. Men with TML reported less physical exercise than men without TML, had a higher consumption of crisp and experienced more testicular discomfort.

Developing TML in testicles appears in general independent of lifestyle factors such as food and soft drink consumption, alcohol, BMI and smoking.

Secondarily, a total of 43 men were diagnosed with cancer (2.8%), and the presence of TML was significantly associated with cancer compared to no TML (5.6% vs. 2.4%) (*p* = 0.011). TML has previously been suggested to be a pre‐malignant condition (Derogee *et al*., 2001; Wang *et al*., 2015). In our study, testicular cancer was associated with TML; however, the study was not designed to elucidate a possible causal link.

Seminoma was the most frequent cancer subtype in both groups. TML has been suggested associated with seminoma (McDonald *et al*., 2012; Sharmeen *et al*., 2015; Pedersen *et al*., 2016). Of the 29 seminomas, a total of 23 (79.3%) men did not have TML and six (20.7%) were diagnosed with TML. This observation questions the suggested association between TML and seminoma.

Additionally, we found that TML may develop over time, as 30 out of 197 men were not diagnosed with TML at their first US investigation. In general, men with TML had more US investigations performed than men without TML, which was expected because of follow‐up in these patients.

### Strengths and weaknesses of the study

A strength of this study was the use of a clinical radiological database for registration of the TML diagnosis. Furthermore, because of the Danish civil registration number, it was possible to link data with biopsies performed and cancer subtype. We investigated a wide range of self‐reported health and scrotal symptoms, which has not previously been reported in men with TML. This study enjoyed a relative high response rate, which reduced the risk of selection bias. Still, one‐third did not participate which may lead to a selection bias associated with having the diagnosis TML. However, as the proportion of TML in responders was the same in the study base, the potential selection bias probably may be ignored.

The questionnaires were used in a paper version and not as a web‐based version, which may have increased the response rate, especially among elderly men. The questionnaire were mailed to men who had an US investigation of the scrotum in the period 2004–2013, and this might have introduced recall bias from some of the responders. Some may have forgotten about the experience of symptoms, and a handful of men phoned because they have elapsed receiving an US investigation in our department. However, in all questions concerning lifestyle, we asked the men how many cigarettes they smoked, and what they have been eating and drinking during the last couple of months, hence recall bias was limited.

### Comparison with other findings

Our study is the first to investigate health in men with TML. ESUR provided in 2015 a guideline (Richenberg *et al*., 2015) suggesting men with TML and additional risk factors, such as family history of testicular cancer, testicular atrophy, maldescent and orchidopexy should be advised to participate in annual US follow‐up until aged 55 years. We did not find any additional risk factors to be considered when offering men with TML yearly US follow‐up. Men with TML reported significantly less physical exercise than men without TML. A review from 2015 investigated the exercise, testicular diseases and reproductive function concluding that the impact of exercise in male reproductive functions remains controversial (Gomes *et al*., 2015). Our data suggest that there may be an association.

Overall, developing TML in testicles appears not to be influenced by lifestyle factors. However, men with TML consumed more crisp and popcorn than men without TML. Crisp and popcorn contains acrylamide, and it is known for its potential health hazards. A study from 2003 investigated the concentration of acrylamide in crisps and found the highest concentration in potato crisps but also potato chips and popcorn had a high concentration (Murkovic, 2004). In some studies, rodent acrylamide has been shown to generate tumours in multiple organs, including mesotheliomas of the testicles (Johnson *et al*., 1986; Friedman *et al*., 1995; Klaunig, 2008). In a study from 2013, the intake of acrylamide was investigated in 10 European countries, and on average, Denmark and the UK had the highest dietary self‐reported intake of acrylamide (Freisling *et al*., 2013). Our data may suggest that further studies are needed in this area, for example, investigating asymptomatic men.

In our department, men with TML were more often US investigated than men without TML because men with TML were invited to participate in the follow‐up programme with scans every 6th month in a period of 2 years. Testicular pain was the most frequent referral reason to US investigation, and men with TML tended more often to have testicular pain than men without. Thus, another reason why men with TML are investigated more often could be the experience of pain in the scrotum.

Patients with genetic disorders like McCune–Albright syndrome, Down syndrome and Klinefelter syndrome appear to have very high frequencies of TML. A recent review did not find TML in patients with McCune–Albright or Down syndrome to be associated with development of testicular cancer (Pedersen *et al*., 2016). It should be noted, however, that males with Down syndrome generally have a higher risk of testicular cancer (Patja *et al*., 2006). Furthermore, a recent study did not find increased development of testicular cancer in Klinefelter patients (Accardo *et al*., 2015). Instead, the association between TML and genetic abnormalities may indicate TML as part of a degenerative process of the testis.

In some studies, mothers smoking during pregnancy have been associated with testicular cancer in the male offspring (Kaijser *et al*., 2003; Pettersson *et al*., 2004). We found an insignificant tendency that men with TML had been more exposed to maternal smoking than men without TML.

Wiren *et al*., (2014) found that increasing height was associated with increased risk of testicular cancer. Similarly a meta‐analysis from 2010 (Lerro *et al*., 2010) found height per 5‐cm increase (OR 1.13) to be associated with testicular germ cell tumour. No difference in height between men with or without TML was seen in our study.

## Conclusion

Development of TML was not associated with classic lifestyle factors such as alcohol consumption, smoking habits or mothers smoking during pregnancy. Also, age and height could not be linked to presence of TML. We did find, however, that men with TML experienced less physical activity and consumed more crisp than men without TML. As ingestion of crisps has potential carcinogenic effect (acrylamide), this finding needs confirmation in a separate study.

## Conflict of Interest

The authors have no conflicts of interest to declare.

## Authors’ Contributions

MRP, HM, SSR and PV were involved in study conception and design. MRP, MMJ and PV were involved in study execution and acquisition of data. MRP, HM and PV contributed to data analysis and interpretation. MRP, HM, PV, PJO, SRR drafted the manuscript. All authors provided substantial intellectual contribution and approved the final version of the manuscript.

## Supporting information


**Appendix S1** Information of age distribution and other characteristics of men with and without TML.

**Appendix S2** Patient's soft drink and food consumption.
